# Eine nicht alltägliche tumorassoziierte Thrombose

**DOI:** 10.1007/s00108-022-01272-5

**Published:** 2022-02-23

**Authors:** Rosemary Poulose, Anselm A. Derda, Mohamed Omar, Christian von Falck, Florian Länger, Jochen Tillmanns, Adrian Groh, Johann Bauersachs, L. Christian Napp

**Affiliations:** 1 Klinik für Psychiatrie, Sozialpsychiatrie und Psychotherapie, Medizinische Hochschule Hannover, Hannover, Deutschland; 2 Klinik für Kardiologie und Angiologie, Medizinische Hochschule Hannover, Carl-Neuberg-Str. 1, 30625 Hannover, Deutschland; 3 Klinik für Unfallchirurgie, Medizinische Hochschule Hannover, Hannover, Deutschland; 4 Institut für Diagnostische und Interventionelle Radiologie, Medizinische Hochschule Hannover, Hannover, Deutschland; 5 Institut für Pathologie, Hannover Medical School, Hannover, Deutschland

**Keywords:** Paraneoplastisches Syndrom, Antikoagulation, Exostose, Chondrosarkom, Knochentumor, Paraneoplastic syndrome, Anticoagulation, Exostosis, Chondrosarcoma, Bone tumor

## Abstract

Es wird über den Fall einer tiefen Beinvenenthrombose berichtet, bei dem im Rahmen der Diagnostik ein zuvor nicht bekanntes Chondrosarkom entdeckt wurde. In der körperlichen Untersuchung zeigte sich am rechten Knie dorsal eine feste, nicht verschiebliche Raumforderung, die in der Sonographie als zystische Formation imponierte. Röntgen, Computertomographie und Kernspintomographie zeigten typische Befunde einer sekundär entarteten kartilaginären Exostose, die sehr wahrscheinlich über lokale Kompression und ggf. über paraneoplastische Mechanismen die Entstehung der Thrombose begünstigt hatte. Nach Resektion des Tumors wurde die präoperativ begonnene Antikoagulation fortgeführt. In Zusammenschau aller Befunde wurde die endgültige Diagnose eines hochdifferenzierten sekundären Chondrosarkoms mit Thrombose der Poplitealvene gestellt.

## Abstract (en)

Here, we report on a patient with deep vein thrombosis of the right leg, in whom diagnostic work-up revealed a previously unknown chondrosarcoma of the tibia. Physical examination revealed a firm, nondisplaceable mass on the dorsal side of the right knee that appeared as a cystic formation on ultrasound. X‑ray, computed tomography, and magnetic resonance imaging were consistent with chondrosarcoma, which had likely provoked the thrombosis by local compression or paraneoplastic mechanisms. After resection of the tumor, anticoagulation was continued. In a review of all findings, a final diagnosis of highly differentiated chondrosarcoma with thrombosis of the popliteal vein was made.

## Anamnese

Eine 72-jährige Patientin wurde aufgrund einer paranoiden Schizophrenie seit 3 Monaten stationär in der Psychiatrie behandelt. Die Patientin war zwar teilweise auf dem Stationsflur mobil, aber aufgrund nicht ausreichender Mobilität wurde eine Thromboseprophylaxe mit subkutanem niedermolekularem Heparin verordnet. Diese wurde von der Patientin im Rahmen eines ausgeprägten Vergiftungswahns verweigert. Zur Behandlung der Schizophrenie wurde nach § 1906a Abs. 2 Bürgerliches Gesetzbuch (BGB) eine Zwangsmedikation mit Paliperidon zunächst wöchentlich und dann alle vier Wochen als intramuskuläres Depotpräparat appliziert. Hierunter besserte sich das klinische Bild, und die Mobilität der Patientin nahm zu. Die Thromboseprophylaxe wurde seitens der Patientin weiterhin nicht akzeptiert. Drei Monate nach Aufnahme klagte die Patientin über plötzlich aufgetretene Schmerzen und Schwellung im rechten Bein. Luftnot oder Angina pectoris lagen nicht vor, ebenso kein Schwindel, Palpitationen oder Bewusstlosigkeit. Weitere Vorerkrankungen waren nicht bekannt, ebenso kein Nikotinkonsum und keine Allergien. Die Familienanamnese war bezüglich Tumorerkrankungen unauffällig, und eine B‑Symptomatik wurde glaubhaft verneint.

## Klinischer Befund

**Figure 1:**
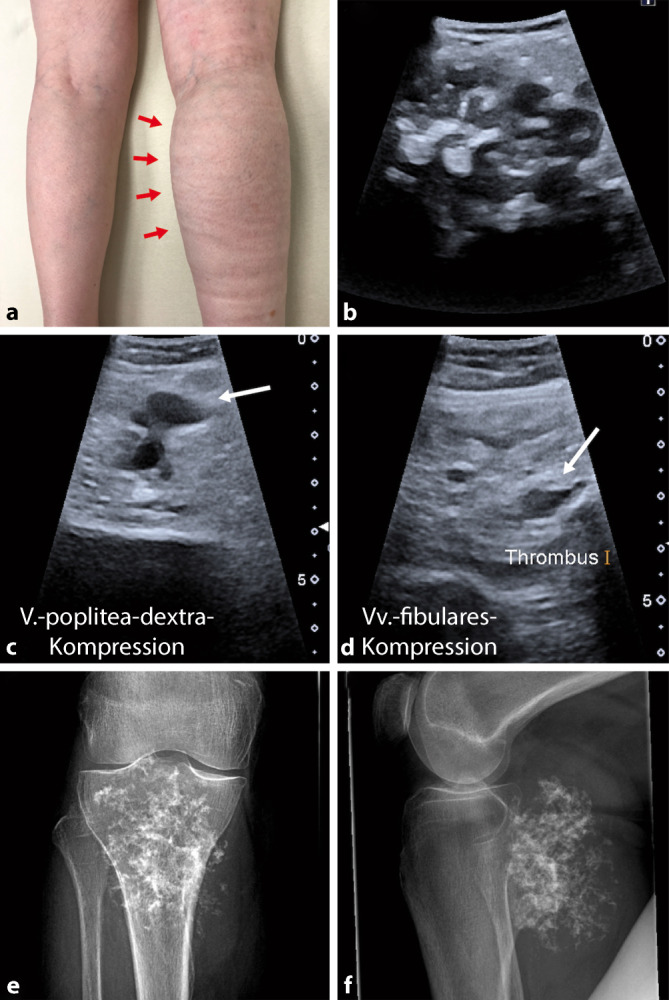

In der körperlichen Untersuchung zeigte sich eine Schwellung des gesamten rechten Unterschenkels sowie des rechten Fußes (Abb. 1a). Die Schwellung ging am Knie dorsal in eine feste, nicht verschiebliche Raumforderung über. Periphere Durchblutung, Motorik und Sensibilität waren intakt. Die Beweglichkeit im rechten Knie war geringgradig reduziert (Extension/Flexion 0‑0-110°). Payr-Zeichen (Fußsohlendruckschmerz) und Meyer-Zeichen (Wadendruckschmerz) als klinische Thrombosezeichen waren negativ.

## Diagnostik

Es wurde zunächst eine Laboruntersuchung veranlasst, die erhöhte D‑Dimere (1,58 mg/l; Normwert 0–0,5 mg/l) ergab. Die Sonographie zeigte als Korrelat der nicht verschieblichen Raumforderung eine zystisch anmutende Formation in der rechten Kniekehle mit einer Ausdehnung von ca. 8 × 5 cm (Abb. 1b). Die Duplexsonographie bestätigte den klinischen Verdacht einer Thrombose, die von der rechten Vena poplitea bis in das mittlere Drittel der Venae fibulares zog (Abb. 1c,d).

## Diagnose: Thrombose der Poplitealvene

Zur Behandlung der Thrombose wurden zunächst eine therapeutische Antikoagulation mit Tinzaparin s.c. (gewichtsadaptiert 10.000 IE pro Tag) sowie ein Kompressionsverband des Beins begonnen, welche von der Patientin nun akzeptiert wurden. Nach fünf Tagen wurde die Antikoagulation bei laborchemisch normaler Nierenfunktion auf Edoxaban (60 mg pro Tag) umgestellt.

Zur weiteren Abklärung der Raumforderung erfolgte eine Röntgenuntersuchung des rechten Kniegelenks in zwei Ebenen. Diese zeigte einen Tumor der poplitealen Weichteile mit „popcornartigen“ Transparenzminderungen (Abb. 1e,f), der sich röntgenologisch nicht eindeutig vom posterioren Tibiaknochen abgrenzen ließ.

**Figure 2:**
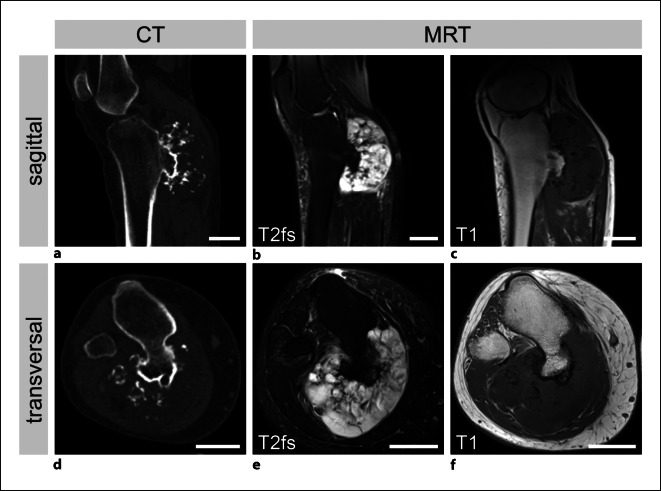

**Figure 3:**
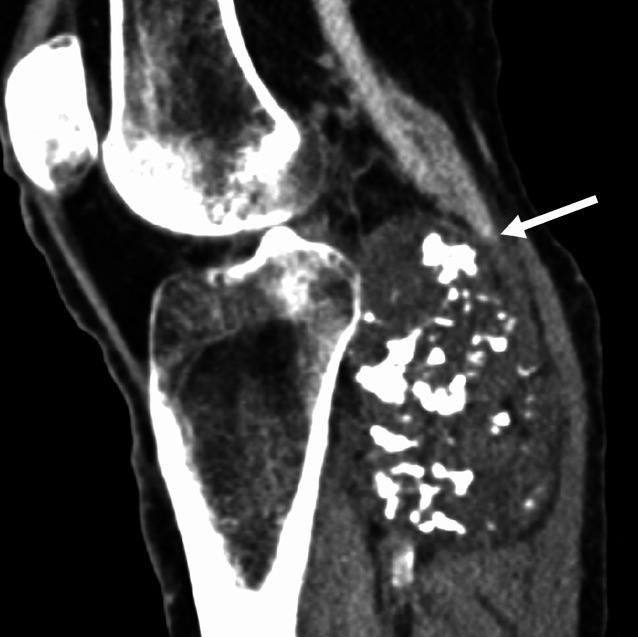

Eine Computertomographie (Abb. 2a, d) mit venöser Kontrastmittelphase wies die Markraumkontinuität der Raumforderung und eine angrenzende chondroide Matrixverkalkung nach. In der ergänzenden Kernspintomographie (Abb. 2b,c,e,f) zeigte sich die Raumforderung (7,2 × 5,1 × 8,8 cm) ebenfalls mit Markraumkontinuität der Basis sowie einem typischen chondroiden Signalverhalten der Kappe mit kräftiger Hyperintensität in den flüssigkeitssensitiven Sequenzen. Die Tumorformation komprimierte die Vena poplitea beginnend am P2-Segment (Abb. 3).

## Diagnose

- Thrombose der Poplitealvene und sekundär entartete kartilaginäre Exostose (Chondrosarkom) der Tibia.

## Therapie und Verlauf

**Figure 4:**
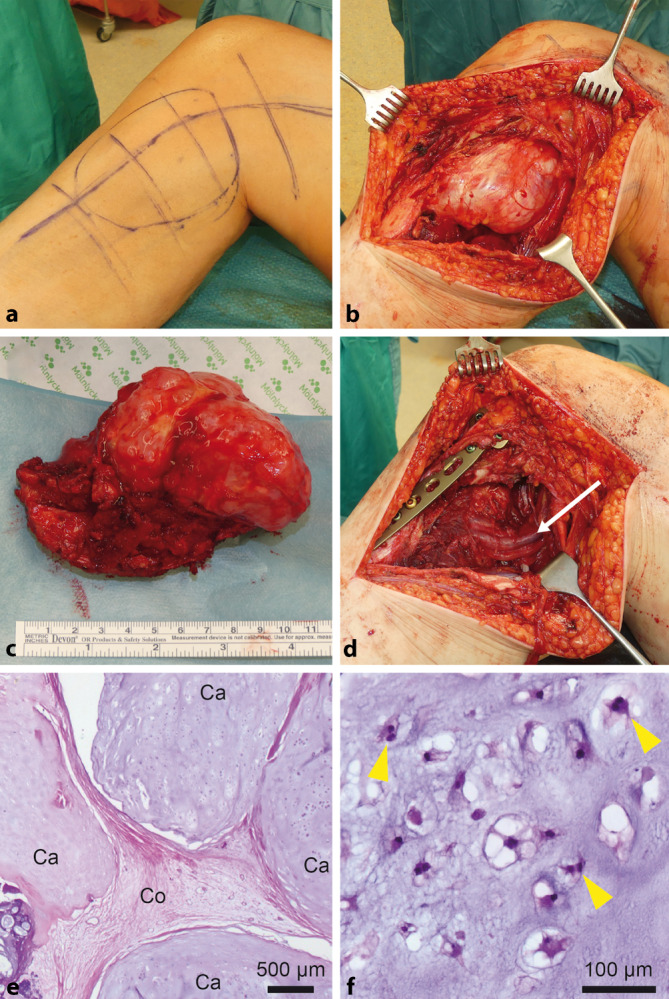

Nach Beschluss der interdisziplinären Tumorkonferenz wurde die Indikation zur operativen Resektion gestellt. In Anbetracht der bildmorphologischen Darstellung war eine vorhergehende bioptische Abklärung nicht erforderlich, da sich hieraus keine Änderung des therapeutischen Vorgehens ergeben hätte: Die Bildgebung hatte typische Befunde eines Osteochondroms gezeigt, mit einer maximalen Ausdehnung der Knorpelkappe von 35 mm. In der vorliegenden Konstellation war von einem Chondrosarkom auszugehen (s. unten), für das die Indikation zur primären intraläsionalen oder marginalen Resektion besteht. Es erfolgte eine marginale Resektion in Intubationsnarkose mit Neurolyse des Nervus tibialis und Präparation der Poplitealgefäße (Abb. 4a–d). Intraoperativ zeigte sich die Vena poplitea thrombosiert. Durch die Abtragung der Exostose mit konsekutiver Destabilisierung der dorsalen Kortikalis erfolgte bei anzunehmender Incompliance der Patientin zur Ausführung einer Teilbelastung eine winkelstabile Plattenosteosynthese (4,5 mm Locking Compression Plate, Depuy Synthes, Tuttlingen, Deutschland).

Die histopathologische Untersuchung des Resektats zeigte eine lobulär organisierte, wechselnd zellreiche Knorpelmatrix mit geringer Atypie und vermehrt zweikernigen Zellformen sowie verdrängendem Wachstum in die periossäre Kollagenmatrix (Abb. 4e,f). Die histologischen Kriterien erlauben jedoch für sich genommen meist keine zuverlässige Abgrenzung zwischen einem Osteochondrom und einem sekundären Chondrosarkom [1]. Daher ist die Dicke der Knorpelkappe das wesentliche diagnostische Kriterium. Ab einer Dicke von 20 mm ist von einem Chondrosarkom auszugehen; im Falle unserer Patientin betrug diese mehr als 30 mm. Somit wurde nach Bildgebung, operativen und histologischen Befunden die endgültige Diagnose eines hochdifferenzierten sekundären Chondrosarkoms auf dem Boden einer kartilaginären Exostose mit Thrombose der Poplitealvene gestellt.

**Figure 5:**
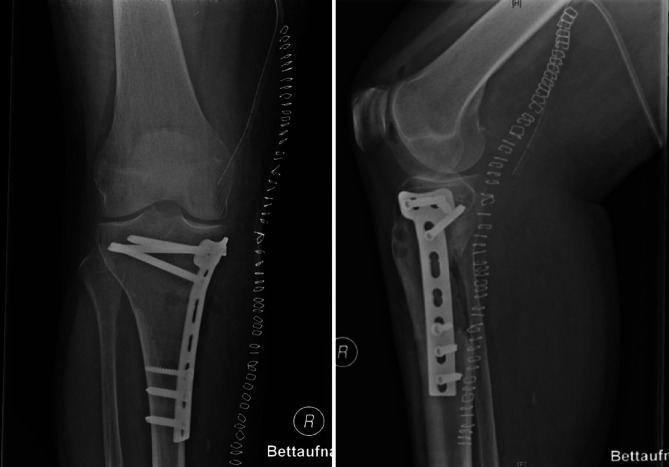

Die Wundheilung gestaltete sich regelrecht und die Mobilisation erfolgte beschwerdeadaptiert. In der postoperativen Bildgebung zeigte sich eine regelrechte Plattenlage (Abb. 5). Die Patientin wurde in gutem Allgemeinzustand und mit trockenen und reizlosen Wundverhältnissen entlassen. Bei der Entlassung zeigten sich die periphere Durchblutung, Motorik und Sensibilität intakt; die Patientin war in Stand und Gang mobil. Die zur Aufnahme führende Schizophrenie ist unter der Depottherapie im Rahmen einer Residualsymptomatik weiterhin gut kontrolliert, der Vergiftungswahn nicht mehr existent und die Patientin nimmt regelmäßig die verordnete Medikation zu sich. Es wurde ein Wiedervorstellungstermin in der angiologischen Sprechstunde vereinbart. In Annahme eines komplikationslosen Verlaufs ist geplant, die Antikoagulation nach sechs Monaten zu beenden.

## Diskussion

Wenn eine Thrombose diagnostiziert wird, liegt in ca. 15 % der Fälle ein bekanntes und in ca. 3–15 % der Fälle ein nicht bekanntes Malignom vor [2]. Zu den häufigsten Krebserkrankungen, die mit einem hohen Risiko für venöse Thromboembolien vergesellschaftet sind, gehören Malignome des Gehirns, des Pankreas, der Lunge, und des Gastrointestinaltrakts [3]. Die Leitlinien der Arbeitsgemeinschaft der Wissenschaftlichen Medizinischen Fachgesellschaften empfehlen neben einem Basislabor ein individuelles Vorgehen und zunächst die Komplettierung der alters- und geschlechtsspezifischen Vorsorgeuntersuchungen, ggf. inklusive eines Röntgenbilds des Thorax und einer Sonographie des Abdomens [2]. Der Mechanismus der tumorassoziierten Thrombusbildung ist multifaktoriell und auf zelluläre und/oder humorale Prozesse zurückzuführen. Tumoren können weiterhin zu Kompression bzw. Pelottierung von Gefäßen führen, was aus mechanischer Ursache die Entstehung einer Thrombose begünstigt.

Die Virchow-Trias beschreibt die wesentlichen Faktoren, die eine Thromboseentstehung begünstigen: Hämodynamik, Hyperkoagulabilität und Endotheldysfunktion. Die Kompression von Blutgefäßen durch Tumoren wie in diesem Fall kann eine venöse Stase hervorrufen. Produkte von Tumorzellen können sowohl eine endotheliale Dysfunktion als auch Hyperkoagulabilität verursachen [4].

Kartilaginäre Exostosen (syn. Osteochondrom) sind gutartige Knochentumoren, die entarten können und dann als sekundäres Chondrosarkom bezeichnet werden. Chondrosarkome machen etwa 20 % der malignen Knochentumoren aus [5]. In seltenen Fällen wurde berichtet, dass sie in Gefäße infiltrieren und metastasieren, wodurch thrombembolische Ereignisse entstehen können [6]. Zu den wichtigsten neurovaskulären Strukturen, die durch die Fossa poplitea verlaufen, gehören die Arteria und Vena poplitea, der Nervus tibialis und der Nervus fibularis communis. Im Fall unserer Patientin führte das von der proximalen Tibia ausgehende große Chondrosarkom zu einer Kompression der Vena poplitea. Nach marginaler Resektion des Chondrosarkoms und somit „kausaler“ Therapie erscheint eine zeitliche Begrenzung der Antikoagulation vertretbar.

## Fazit für die Praxis

Das Auftreten einer Thrombose kann das erste Anzeichen einer Tumorerkrankung sein. Ungewöhnliche Befunde in der Primärdiagnostik (insbesondere: Anamnese, körperliche Untersuchung, Ultraschall) sollten immer Anlass zu weiterführender Diagnostik und interdisziplinärer Bewertung geben. Wenn ein auslösendes Ereignis nicht identifiziert werden kann, sollte an das Vorliegen einer Tumorerkrankung gedacht werden. Zur Sarkomdiagnostik im engeren Sinne dienen bildgebende Verfahren (Röntgen, Computertomographie, Kernspintomographie), und die Diagnose sollte soweit möglich immer bioptisch gesichert werden.
